# Supplementary material for: Transcriptomic profiling of shed cells enables spatial mapping of cellular turnover in human organs
Source: Mol Syst Biol. 2025 Oct 2;21(12):7. doi: 10.1038/s44320-025-00154-w (PMC12673112; doi:10.1038/s44320-025-00154-w)
Supplement: Supplementary file 5 — Expanded View Figures [file 44320_2025_154_MOESM5_ESM.pdf]

Expanded View Figures

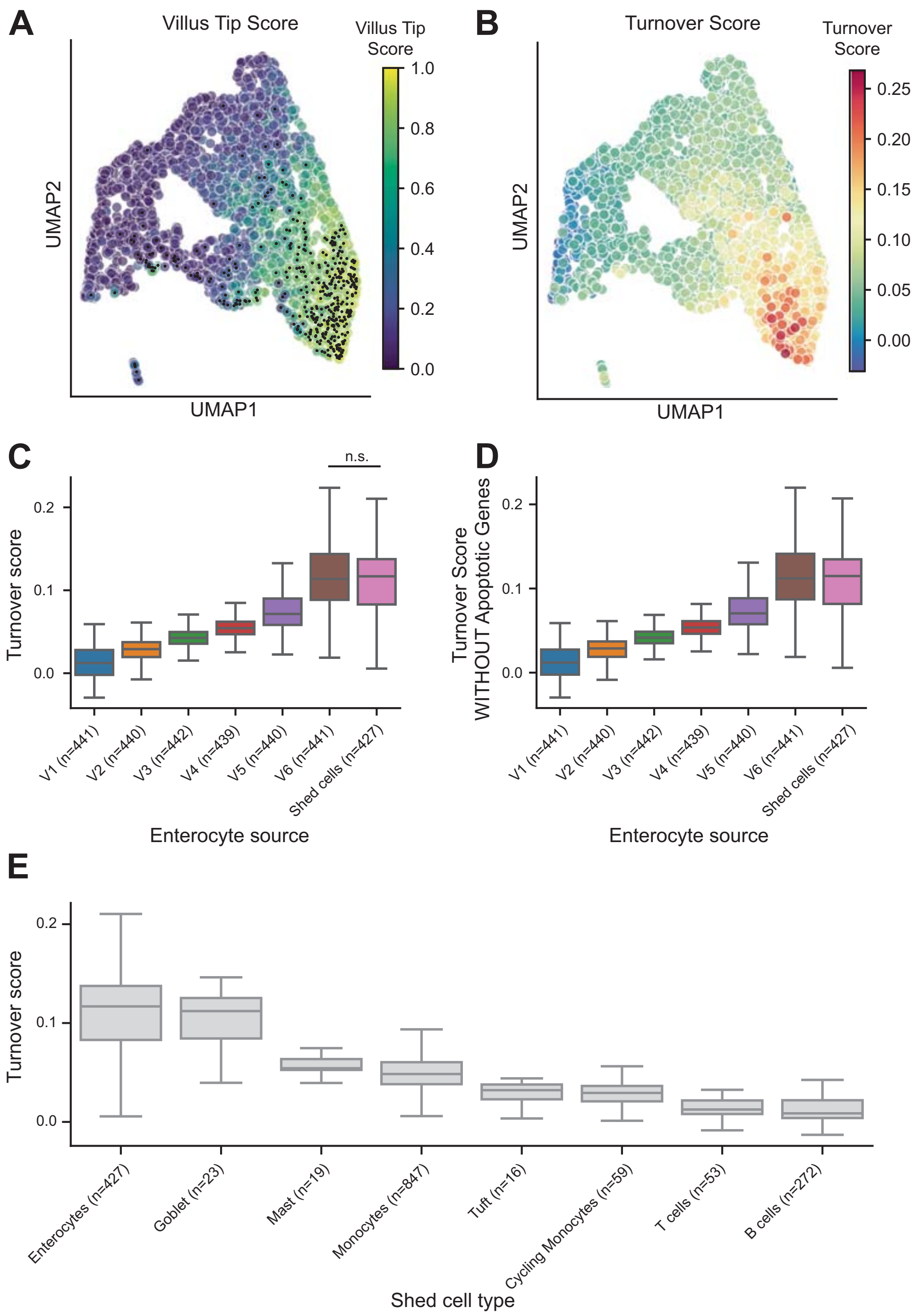

Figure EV1. Turnover scores of single cells from mouse small intestine.

(**A**) UMAP displaying tissue enterocytes and shed cells, with shed cells marked with black dots. (**B**) UMAP colored by Turnover score. (**C**) Turnover score of each zone, apoptotic genes included, there is no significant difference between V6 and shed-cells ($P = 0.1$, Wilcoxon rank-sum test). (**D**) Turnover score of each zone and shed cells without apoptotic genes. (**E**) Turnover score of all shed cells types from Bahar Halpern dataset (Bahar Halpern et al, 2023). Box plots show the median as the center line, boxes span the 25th–75th percentiles, whiskers extend up to 1.5 IQR, outliers are not shown.

 © The Author(s)

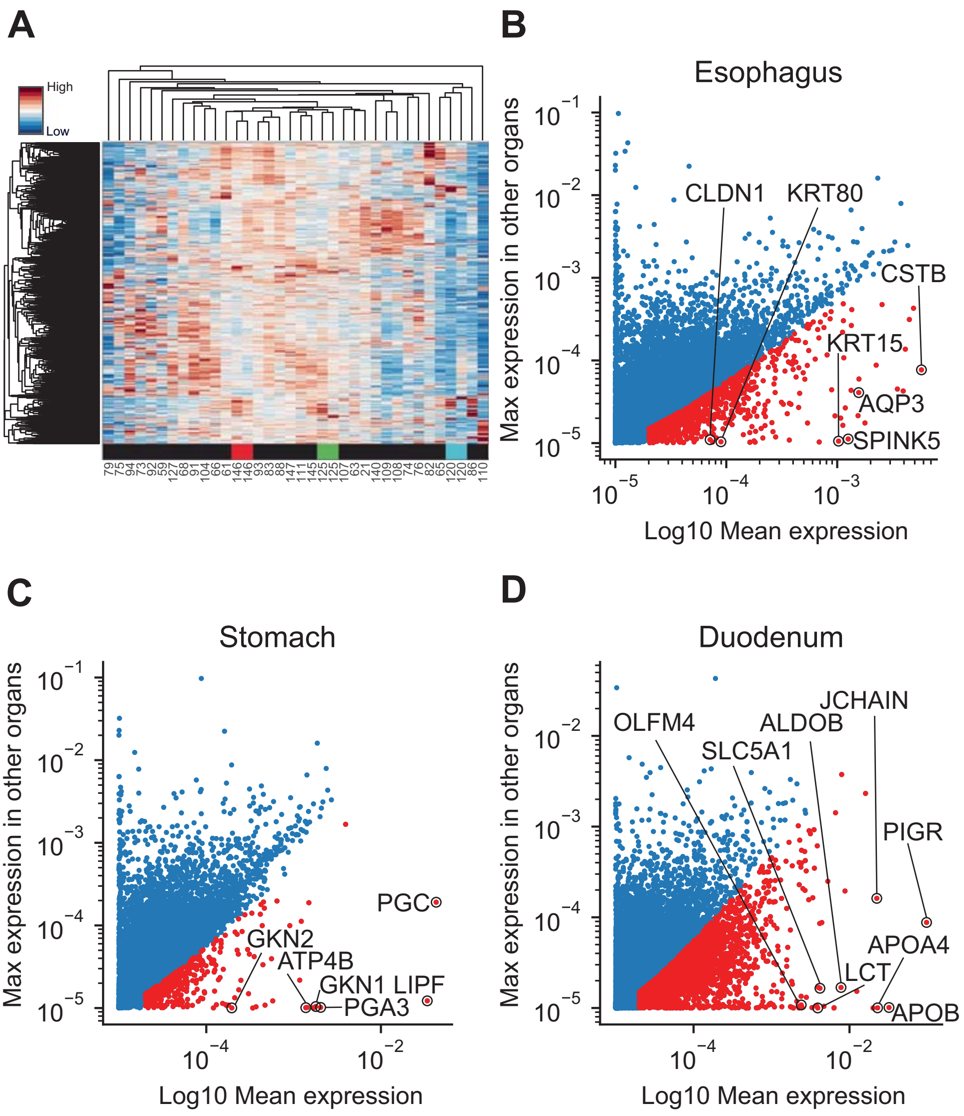

Figure EV2. Reproducibility and marker stratification of NGT fluids.

(**A**) Clustergram of NGT samples shows reproducibility across patients. Every column represents a sample and every row represents a gene. Colors denote biological repeats obtained at 10–15 min intervals. (**B–D**) Marker gene selection of the analyzed tissues—Esophagus (**B**), Stomach (**C**), and Duodenum (**D**). Red dots denote genes with maximal expression above 1e-5 and fold-change above 2 compared to the remaining two organs. Representative genes are highlighted. A pseudo-number of 1e-5 was added to the data.

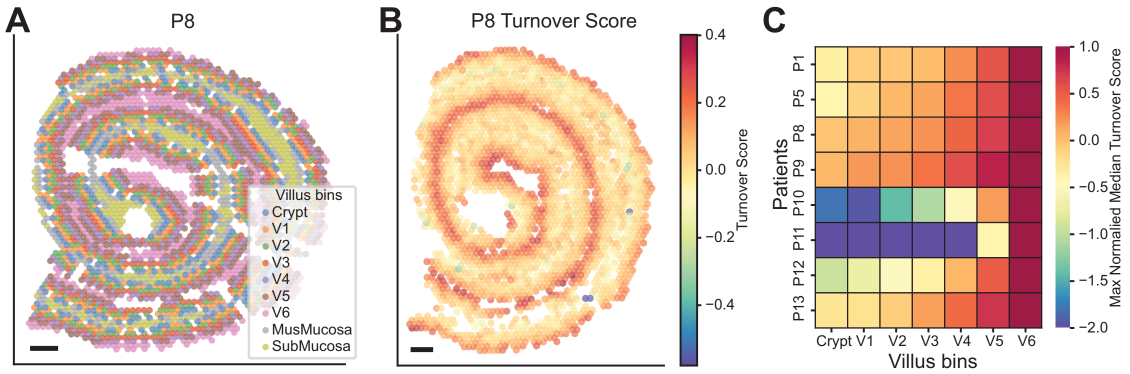

Figure EV3. Spatial turnover maps of the human small intestine.

(**A**) Annotated Visium dataset of the human small intestine from Harnik et al (Harnik et al, 2024). (**B**) Visium data colored by turnover score. Scale bars in (**A**, **B**) are 500 µm. (**C**) Average turnover score of crypt-villus zones for the 8 patients analyzed in Harnik et al (Harnik et al, 2024). Values lower than −2 were set to −2.

 © The Author(s)

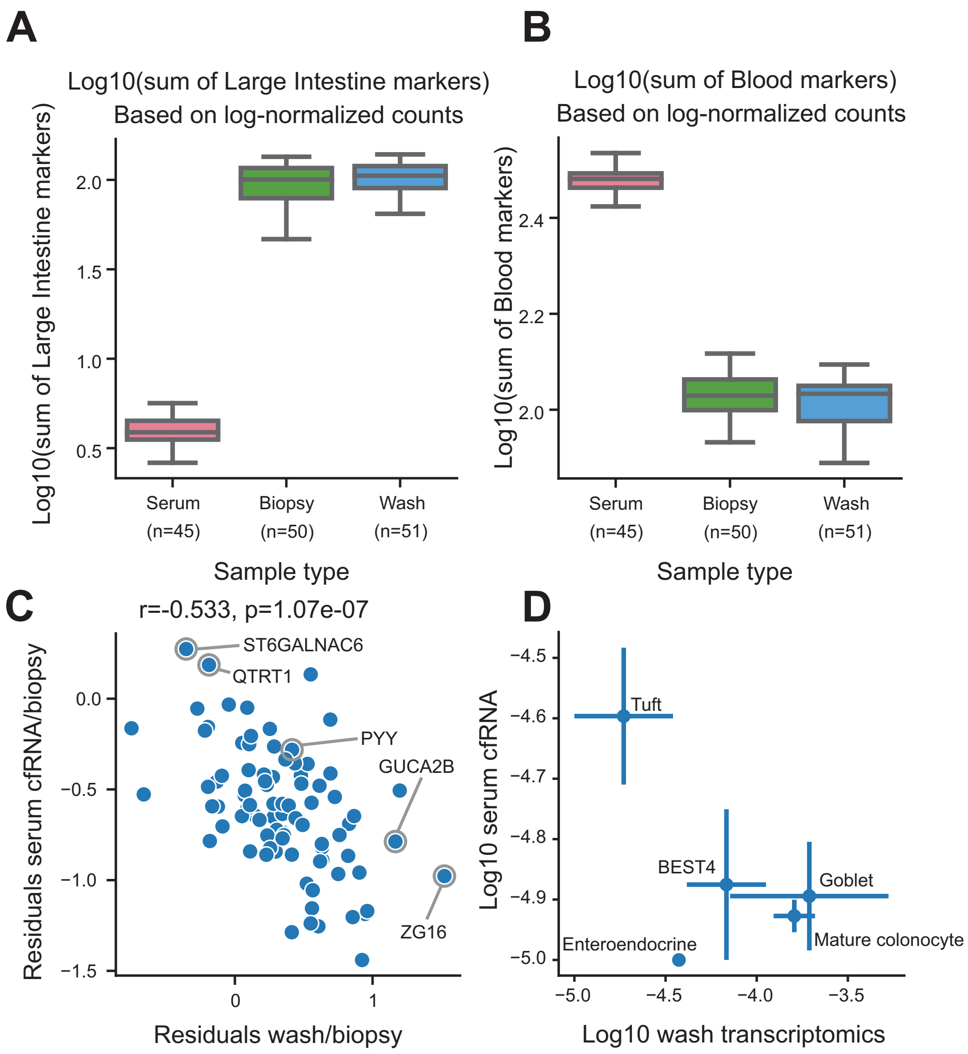

Figure EV4. Luminal shedding is different from basolateral shedding.

(**A**) Sum of Large Intestine markers (log-normalized counts) of serum, biopsy and wash. (**B**) Sum of Blood markers of serum, biopsy and wash, serves as a positive control for (**A**). (**C**) Scatter plot of residuals of expressed genes in both wash and biopsy, representative genes are shown. (**D**) Mean large intestine cell type markers expression in serum cfRNA and wash transcriptomics. The number of genes were 22 in mature enterocytes, 6 in Goblet cells, 4 in Tuft cells, 2 in BEST4 cells and 1 in Enteroendocrine cells. Box plots show the median as the center line, boxes span the 25th–75th percentiles, whiskers extend in (**A–C**) extend up to 1.5 IQR, outliers are not shown. Whiskers in (**D**) represent SEM.
